# Prolonged neonatal jaundice

**DOI:** 10.1186/1824-7288-41-S2-A36

**Published:** 2015-09-30

**Authors:** Antonietta Giannattasio, Giusy Ranucci, Francesco Raimondi

**Affiliations:** 1 Department of Translational Medical Science-Section of Neonatology, University Federico II, Naples, Italy

## 

Prolonged neonatal jaundice is defined as a jaundice lasting more than 14 days of life in the full-term infants [1,2]. Etiologically it is helpful to distinguish jaundice related to unconjugated (indirect) or conjugated (direct) hyperbilirubinemia. A prolonged unconjugated hyperbilirubinemia may be related to breastfeeding or to some pathological conditions as hemolytic diseases (due to Rh or AB0 incompatibility, or G6PD deficiency), congenital hypothyroidism, urinary infection, Crigler-Najjar or Gilbert syndromes [1,2]. Conjugated hyperbilirubinemia (cholestatic jaundice) is never physiologic. It affects 1/2500 live births and it should be suspected in all jaundiced infants with light stools and dark urine [3,4]. Delayed referral of cholestatic neonates is still a significant problem. To promote early diagnosis of cholestasis, it is recommended that any infant who remains jaundiced beyond age 2 to 3 weeks (for breastfed infants who can be monitored and who have an otherwise normal history and physical examination) should have the serum bilirubin level fractionated [5].

The differential diagnosis of cholestasis is extensive, and early recognition is essential to ensure timely treatment and optimal prognosis [6]. Developments in molecular genetic techniques have enabled the identification of causative genes, which has improved diagnostic accuracy for patients [7].

In case of neonatal cholestasis, the first step should be the assessment of coagulation and urgent parenteral vitamin K administration in case of coagulopathy and the exclusion of life-threatening conditions or disorders requiring urgent specific medical (eg. NTBC in tyrosinemia, elimination diet in galactosemia and ereditary fructose intolerance) and surgery treatment. Biliary atresia is the most frequent single cause of neonatal cholestasis and affected infants appear otherwise healthy and grow normally [8]. Early performance of a hepatoportoenterostomy in the first 45 days of life to restore bile flow and lessen further damage to the liver is thought to optimize outcome [9].

Infants admitted in NICUs have a rate of cholestasis higher than that reported in the general population of live births; in most cases, cholestasis is associated to multiple risk factors and shows a favorable outcome [10].

Long-term cholestasis determines malnutrition, psychomotor development delayed and immune deficiency. So that even when specific treatment is not available, infants who have cholestasis benefit from early medical management and nutritional support for malabsorption and vitamin deficiency [8].
